# Transplant of insulin‐like growth factor‐1 expressing bone marrow stem cells improves functional regeneration of injured rat uterus by NF‐κB pathway

**DOI:** 10.1111/jcmm.13574

**Published:** 2018-03-07

**Authors:** Lei Wang, Mengnan Yang, Minfei Jin, Yuelin Wu, Tao Zheng, Shengyi Gu, Xiaolin Hua

**Affiliations:** ^1^ Department of Obstetrics and Gynecology Xinhua Hospital Shanghai Jiaotong University School of Medicine Shanghai China

**Keywords:** bone marrow stem cell, IGF‐1, IL‐10, p50, uterus injury

## Abstract

To investigate the potential beneficial effect of insulin‐like growth factor‐1 (IGF‐1) in BMSC transplantation therapy of uterus injury and the underlying molecular mechanisms, rat BMSCs were isolated and cultured. The relative expressions of IGF‐1 and IL‐10 were determined by RT‐PCR and immunoblotting. The secretory IL‐10 and released E2 were measured using ELISA kits. The relative vWF and α‐SMA expressions were determined by immunohistochemistry. The direct binding of NF‐κB subunit p50 with IL‐10 promoter was analysed by chromatin immunoprecipitation assay. The regulation of IL‐10 expression by p50 was interrogated by luciferase reporter assay. Our data demonstrated that IGF‐1 expression in BMSCs induced IL‐10 expression and secretion, which was further enhanced by E2‐PLGA. IGF‐1 overexpression improved BMSCs transplantation therapy in rat uterus injury. We further demonstrated that both inhibition and knockdown of p50 abolished IGF‐1‐induced expression and secretion of IL‐10 in BMSCs, which consequently compromised the IGF‐1 conferred therapeutic benefits against uterus injury. Furthermore, we elucidated that p50 regulated IL‐10 expression via direct association with its promoter. Our data suggested that transplantation of IGF‐1 overexpressing BMSCs improved functional regeneration of injured uterus by inducing IL‐10 expression and secretion via activation of NF‐κB signalling.

## 1 INTRODUCTION

Uterine endometrium is the inner epithelial layer along with its mucous membrane in the women uterus, which consists of a basal layer and functional layer. The functional layer sloughs and renews in a cyclic manner with menstrual period, whereas the regeneration occurs exclusively in the basal layer.^1^ Any damage related to the basal layer of endometrium severely causes epithelial regenerative disorders, scar formation, fibrosis, intrauterine adhesion (IUA) and consequently engenders amenorrhoea and infertility.^2^ The mainstream clinical remedies include adhesiolysis under hysteroscope with adjuvant oestrogen therapy and post‐surgery placement of intrauterine device or balloon‐expandable stents, which manifests certain curative effect for the mild and moderate cases.^3^ However, the prognosis for the severe cases is unsatisfactory and the incidence of recurrent adhesion up to 20%‐62.5%, and frequently associates with irreversible impairment with respect to the receptivity and fertility.^4^ Therefore, to improve the proliferation and regeneration of endometrial cells, which in turn to reconstruct the endometrial structure and restore the endometrial functioning, is the radical curation for endometrial damage to improve pregnancy rate.

Bone marrow‐derived mesenchymal stem cells (BMSCs) are multipotent stem cell with potential to differentiate into array of mature cell types, which are increasingly clinically exploited for regenerative medicines against complicated tissue damages.^5^ Our previous study demonstrated promising therapeutic effect of minimally invasive BMSCs transplantation in the uterine prolapse rat model.^6^ Likewise, another investigation showed that BMSCs differentiated into endometrial epithelial cells, which promoted proliferation of endometrial stromal cells and accretion of endometrium, and eventually contributed to the elevated pregnancy rate in the endometrial damage animal model.^7^ More importantly, Singh et al proposed the autologous stem cell transplantation as a novel cell‐based therapy for refractory Asherman's syndrome.^8^ Mechanistically, the transplanted BMSCs have the potential to activate the resident uterine endometrial stem cells via autocrine and paracrine pathway and motivate cell interactions to reconstruct the injured uterine endometrium.^9^ Moreover, it is convincing that the endometrial stem cells highly likely originate from BMSCs,^10^ which fosters the hypothesis of the dual function of BMSCs in endometrial repair via both direct differentiation into endometrial epithelial cell and activation of the resident endometrial stem cell. In this study, we inoculated the autologous BMSCs directly into uterine cavity in the endometrial damage rat model to improve endometrial regeneration and restore endometrial functions. However, with the intrinsic limitation that low proportion of stem cells in bone marrow cell population and declined differentiative potency with age,^10^ the therapeutic effect of direct injection of BMSCs is greatly inferior to the control currently, which immediately imposes two critical issues for clinical applications: to improve the BMSCs survival, attachment, proliferation and differentiation; to improve the differentiated epithelial cell proliferation and eventually restoration of endometrium.

Insulin‐like growth factor‐1 (IGF‐1) exists in both endometrial epithelial cells and basal cells with critical physiological functions. In response to the oestrogen stimulations, IGF‐1 binds to its receptor on the epithelial and basal cell surfaces to significantly promote cell proliferation.^11^ Moreover, IGF‐1 suppresses inflammatory reaction and fibrous scar formation via inhibition of inflammatory cytokine release.^12^ IGF‐1 also plays an important role in angiogenesis via up‐regulation of vascular endothelial growth factor (VEGF).^13^ Notably, IGF‐1 also promotes BMSCs proliferation and differentiation into endometrial epithelial cell and stimulates BMSCs migration to damage sites and involves in tissue repair.^14^ Therefore, elevation of secreted IGF‐1 in endometrium might provoke the proliferation and differentiation of BMSCs into endometrial epithelial cell and basal cell, which in turn reinforces the differentiative potential of BMSCs via array of secretory cytokines in a positive feedback loop, and this synergistic effect consequently contributes to endometrial repair and regeneration. In line with this notion, here we established IGF‐1‐overexpressing BMSCs to explore its beneficial effect in treatment of endometrial damage.

Accumulative investigations suggest that oestrogen is indispensable for endometrial regeneration and repair, and the steady and persistent intrauterine oestrogen supply is critical for this processing.^15^ We have previously prepared the sustained release nanoparticles with biodegradable poly(lactide‐co glycolide‐co‐caprolactone, PLGA), which remarkably alleviated pelvic floor dysfunction both in vitro and in vivo with excellent biostability and biocompatibility.^16^ Therefore, we will continuously adopt this strategy in this study to sustain consistent supply of oestrogen to stimulate endometrial regeneration.

## 2 MATERIALS AND METHODS

### 2.1 Primary rat BMSC isolation and culture

The animal study was performed in strict accordance with the guideline of the Animal Care and Use Committee in the Xinhua Affiliated Hospital of Shanghai Jiao Tong University. Rat BMSCs were isolated and cultured as previously described.^9^ In brief, the 2‐month‐old female rats were killed by overdosing ketamine. The femora and tibias were isolated and thoroughly rinsed with phosphate‐buffered saline (PBS, Gibco, Grand Island, NY, USA). The muscle and extraoral tissues were cautiously removed, and the marrow cells in the bone cavities were completely flushed out with low‐glucose Dulbecco's modified Eagle's medium (DMEM, Gibco, USA). The resultant lavage was passed through 100‐μm cell strainer (BD Bioscience, Franklin Lakes, NJ, USA) and centrifuged to collect single cells, which were then resuspended in LG‐DMEM supplemented with 12.5% foetal bovine serum (BSA, Gibco, USA), 1× insulin‐transferrin‐selenium (ITS, Gibco, USA), 10 ng/mL basic fibroblast growth factor (bFGF, Gibco, USA) and 1% penicillin‐streptomycin‐glutamine (PSG from Gibco, USA). The isolated cells were seeded into 100‐mm Petri dish and maintained at 37°C in a humidified incubator supplied with 5% CO2. The fresh medium was replaced every 2 days, and the log‐phase cells of passage 3‐5 were used for the following experiments. Transfection was performed with lipofectamine 2000 unless indicated and in accordance with the manufacturer's instruction.

### 2.2 Stable IGF‐1 transduction

Transduction of BMSCs with IGF‐1 was performed with adenovirus infection. The coding region of rat IGF‐1 was amplified by regular PCR and fused with GFP into the modified adenovirus (Ad) vector, which was referred as Ad‐CMV‐GFP‐IGF‐1 hereafter. The virus particles were packaged in AD‐293 cells by transient transfection for 24 hours, then the medium was replaced and transfectant was consecutively cultured for 10‐14 days. The conditioned medium was collected as virus stock, and plaque forming units were determined by infecting AD‐293 cells. The log‐phase BMSCs were seeded into 6‐well plate and infected with Ad‐CMV‐GFP‐IGF‐1 for 24 hours, and the cells were transferred to 100‐mm Petri dish and subjected to G418 selection for 1 week. The formed colonies were characterized by both immunoblotting and PCR.

### 2.3 Stable p50 shRNA knockdown

The MISSION Lentiviral shRNA kit targeting p50 (SHCLNG‐NM_008689), together with the MISSION Non‐Target shRNA Control (SHC016), was purchased from Sigma‐Aldrich (St. Louis, MO, USA) and packaged into lentivirus following the manufacturer's instructions. The log‐phase BMSCs were seeded into 6‐well plates and infected with p50 shRNA lentivirus particles for 24 hours, and the cells were transferred to 100‐mm Petri dish and subjected to puromycin selection for 1 week. The formed colonies were characterized by both immunoblotting and PCR.

### 2.4 E2‐loaded poly(lactide‐coglycolide‐co‐caprolactone) nanoparticles

The PLGA‐NPs were prepared as described previously,^6^ and double‐emulsion solvent evaporation method (water‐oil‐water) was adopted for the formation of E2‐encapsulated NPs. First, 20 g of PLGA was dissolved in dichloromethane and injected into the E2‐containing inner aqueous phase (W1, 100 μL of PBS). The mixture was emulsified with the polymer solution and homogenized by the high‐speed IKA Ultra‐Turrax homogenizer (IKA, China). The emulsion was added to the second aqueous phase (W2), and multiple emulsion (W1/O/W2) was achieved by homogenization. The organic solvent was then evaporated and copolymer was precipitated and collected by centrifugation. NPs were preserved in liquid nitrogen prior to lyophilization for freeze‐drying.

### 2.5 RT‐PCR

The total RNA was extracted from indicated cells using TRIzol reagent (Invitrogen, Carlsbad, CA, USA) following the manufacturer's instruction. The quantity and quality were determined by Bioanalyzer 2100 (Agilent, Santa Clara, CA, USA). The cDNA was synthesized with the High‐Capacity cDNA Reverse Transcription Kit (ThermoFisher, Waltham, MA, USA). The RT‐PCR was performed with GoTaq Green Master Kit (Promega, Madison, WI, USA) in accordance with the manufacturer's recommendation. The primers were ordered from GENEWIZ, China, and listed as below:

*IGF‐1* Forward Primer: 5′‐AAATCAGCAGCCTTCCAACTC‐3′

*IGF‐1* Reverse Primer: 5′‐GCACTTCCTCTACTTGTGTTCTT‐3′

*IL‐10* Forward Primer: 5′‐AGCCTTATCGGAAATGATCCAGT‐3′

*IL‐10* Reverse Primer: 5′‐GGCCTTGTAGACACCTTGGT‐3′

*p50* Forward Primer: 5′‐GGAGGCATGTTCGGTAGTGG‐3′

*p50* Reverse Primer: 5′‐CCCTGCGTTGGATTTCGTG‐3′

*GAPDH* Forward Primer: 5′‐GGAGCGAGATCCCTCCAAAAT‐3′

*GAPDH* Reverse Primer: 5′‐GGCTGTTGTCATACTTCTCATGG‐3′

The relative expression of target gene was calculated by the 2^−∆∆Ct^ method and normalized to *GAPDH*. RT‐PCR results were also verified by agarose gel electrophoresis, with images shown below respective panels.

### 2.6 Western blot

The cell lysates were prepared in RIPA lysis buffer supplemented with Protease Inhibitor Cocktail and Protein Phosphatase Inhibitor Cocktail (Roche, Upper Bavaria, Germany) on ice. The cell debris was discarded after refrigerated centrifugation, and the supernatant was collected for quantification using the BCA Protein Assay Kit (ThermoFisher, USA). The equal amount of protein sample was resolved by SDS‐PAGE gel and transferred to PVDF membrane on ice. The PVDF membrane was blocked with 5% skim milk in TBST buffer at room temperature for 1 hour and hybridized with indicated primary (anti‐p50, ab32360, Abcam, Cambridge, MA, USA 1:1000; anti‐IGF‐1, ab9572, Abcam, 1:1000; anti‐IL‐10, ab34843, Abcam, 1:1000; anti‐GAPDH, ab9485, Abcam, 1:1000) antibodies at 4°C overnight. After rigorous wash with TBST for 5 minutes by 6 times, the PVDF membrane was incubated with horseradish‐conjugated secondary antibodies (anti‐mouse, ab97046, Abcam, 1:5000; anti‐rabbit, ab6721, Abcam, 1:5000) at room temperature for 1 hour. The free antibodies were completely washed off with TBST for 5 minutes by 6 times, and the protein bands were visualized using the enhanced chemiluminescence kit (ELC, Millipore, Billerica, MA, USA) and images were acquired with ChemiDoc Touch Imaging System (Bio‐Rad, Hercules, CA, USA).

### 2.7 ELISA

The secretory IFG‐1 was determined using the commercial Rat IGF‐1 Quantikine ELISA Kit (MG100, R&D SYSTEMS, USA) in accordance with the manufacturer's instruction. Briefly, the conditioned medium was collected from indicated cell culture and subjected to centrifugation to remove any cell debris. 50 μL of supernatant was mixed with 50 μL of assay diluent into each well of 96‐well plate and incubated at room temperature for 2 hours on a horizontal orbital microplate shaker. The supernatant was aspirated completely, and wells were washed thoroughly with wash buffer for at least 5 times. The substrate solution was added to each well and incubated at room temperature for 30 minutes in dark, and reaction was terminated by 100 μL stop solution. The absorbance at 450 nm was recorded with TECAN Infinite 200 PRO (TECAN, USA) with OD 570 as reference.

### 2.8 Rat uterine horn damage model and BMSC transplantation

The vaginal smears were collected daily in the early morning. In total, 32 female Sprague‐Dawley rats with average body weight 250‐300 g and four consecutive 4‐day oestrus cycles were randomly and equally divided into four groups: sham‐operated group, spontaneous repair group, collagen/BMSCs group, collagen/BMSCs‐IGF‐1. To establish uterine horn damage model, the rats were first anaesthetized and the uterine horns were exposed via a low abdominal midline incision. A segment (1.5 × 0.5 cm) was resected from the horn of the uterine with the side of mesometrium left intact. The collagen scaffolds with or without BMSCs were sutured to replace the excised segments. For spontaneous repair, the uterine horns with complete haemostasis and obvious defects were left open to allow healing without further treatments. For sham operation, the uterine horns were left intact after exposure by an abdominal midline incision. The procedure was illustrated in Figure S1. All rats were intramuscularly administrated with penicillin twice a day for three consecutive days after surgery to prevent systematic infection.

### 2.9 Histological examination

The regenerative region of uterine horn was resected and fixed with 4% paraformaldehyde overnight, dehydrated with graded alcohol and embedded in paraffin. The tissues were sliced into 5 μm sections transversally with microtome (Leica RM2255, German). Tissue sections were then subjected to immunostaining with anti‐α‐smooth muscle actin antibody (α‐SMA, ab5694, 1:200) and anti‐von Willebrand factor antibody (vWF, ab6994, 1: 10000), respectively. The muscle actin density was estimated by the percentage of α‐SMA‐positive area with Image‐Pro Plus software, and capillary vessels were counted under light microscope in at least three random fields.

### 2.10 Statistical analysis

All data in this study were acquired from at least three independent experiments. The results were analysed with SPSS 23.0 and presented as mean ± SD. One‐way analysis of variance (ANOVA) was employed for multiple comparisons followed by Turkey post hoc test. The statistical significances were calculated as *P* values, and *P* < .05 was considered statistically different.

## 3 RESULTS

### 3.1 IGF‐1 expression in BMSCs induces IL‐10 expression and secretion

**Figure 1:**
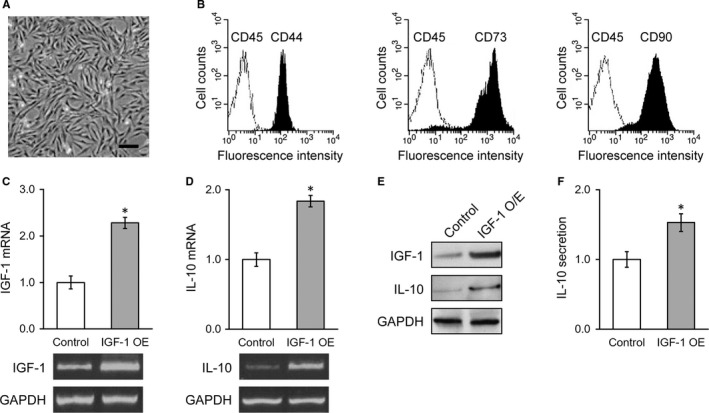
Stable IGF‐1 expression in BMSCs induces IL‐10 expression and secretion. (A) Representative image of cultured BMSCs, scale bar 100 μm. (B) Flow cytometry identified BMSC positive surface markers CD44, CD73 and CD90, and negative surface marker CD45. C and D, mRNA levels of IGF‐1 (C) and IL‐10 (D) in control BMSCs and IGF‐1‐transduced BMSCs, with representative gel images of PCR products shown below the bar graph. (E) Protein levels of IGF‐1 and IL‐10 in control BMSCs and IGF‐1‐transduced BMSCs. (F) IL‐10 levels in media culturing control BMSCs and IGF‐1‐transduced BMSCs. Values are mean ± SD. *P < .05 vs control. IGF‐1, Insulin‐like growth factor‐1; BMSC, Bone marrow‐derived mesenchymal stem cells

First of all, we confirmed the identity of the rat BMSCs (Figure 1A) by flow cytometry analysis, showing that these cells were positive for surface markers CD44, CD73 and CD90, while negative for surface marker CD45 (Figure 1B), as reported previously.^17^ Next, to investigate the potential stimulatory effect of IGF‐1 in promotion the expression and secretion of IL‐10 in cultured BMSCs cells, here we first established stable cell line with overexpression of IGF‐1. The successful overexpression of IGF‐1 in BMSCs was validated by RT‐PCR as shown in Figure 1C. The endogenous expression of IL‐10 was significantly induced by introduction of IGF‐1 with approximate 1.8‐fold increase (Figure 1D), which was consistent with previous report demonstrated that IGF‐1 suppressed inflammation via promoting the secretion of anti‐inflammatory cytokine IL‐10. This phenotype was further confirmed at protein level as shown in Figure 1E, which displayed remarkable up‐regulation of IL‐10 in response to IGF‐1. In view of its intrinsic feature as a secretory cytokine to functioning physiologically, here further examined IL‐10 content in culture medium of IGF‐1 overexpressed BMSCs. Our ELISA results demonstrated that the secretion of IL‐10 was significantly stimulated by IGF‐1 overexpression as well (Figure 1F). Taken together, our results suggested that IGF‐1 overexpression in BMSCs cells remarkably stimulated both expression and secretion of IL‐10, which might consequently contribute to the anti‐inflammatory effect under pathologic conditions.

### 3.2 E2‐PLGA further enhances IGF‐1 expression and IL‐10 secretion in stable IGF‐1‐expressing BMSCs

**Figure 2:**
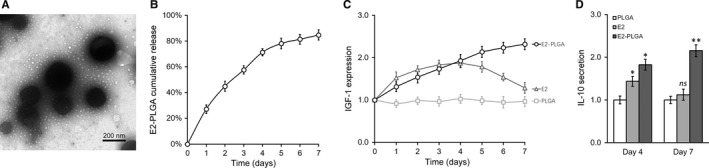
E2‐PLGA further enhances IGF‐1 expression and IL‐10 secretion in stable IGF‐1‐expressing BMSCs. (A) Electronic microscopic image of E2‐PLGA particles. (B) In vitro cumulative release profile of E2‐loaded PLGA. C, IGF‐1‐transduced BMSCs were cultured in the presence of empty PLGA, free E2 without PLGA (E2) or E2‐loaded PLGA (E2‐PLGA), and IGF‐1 expression level was monitored daily through a course of 7 days. (D) IL‐10 levels in different media as described in (C) culturing IGF‐1‐transduced BMSCs were measured on days 4 and 7. Values are mean ± SD. **P < .01, *P < .05, ns—not significant, vs PLGA. IGF‐1, Insulin‐like growth factor‐1; BMSC, Bone marrow‐derived mesenchymal stem cells

The previous study suggested that E2 directly associated with ER‐α, which in turn induced IGF‐1 expression in endometrial epithelium cells and eventually improved injury repair and regeneration.^18^ Here, we encapsulated E2 in PLGA nanoparticles (Figure 2A) to investigate the potential synergistic effect in induction of IGF‐1 expression and secretion in BMSCs cells. We first characterized the controlled releasing behaviour of E2‐PLGA by directly assaying free E2 in regular culture medium up to 7 days using commercial ELISA kit. As shown in Figure 2B, the released percentage of E2 gradually increased during the experimental time span, and around 80% of E2 was free and available in the culture medium. Next, we compared the stimulatory effects between direct administration of E2 with controlled release from E2‐PLGA nanoparticles. The direct dosage of E2 induced acute up‐regulation of IGF‐1 which peaked at day 4 and gradually returned to its original level. However, the IGF‐1 expression in E2‐PLGA‐treated BMSCs cell increased in a sustainable manner and reached the peak level at day 7 (Figure 2C). Notably, the maximum induced expression level of IGF‐1 in E2‐PLGA group was significantly higher than E2 group, which indicated that E2‐PLGA was superior to E2 to induce IGF‐1 expression with respect to either intensity or sustainability (Figure 2D). Therefore, we employed E2‐PGLA and intramuscularly administrated every 7 days to maximize the therapeutic effect of transplanted BMSCs to treat injured uterus hereafter.

### 3.3 IGF‐1 improved BMSCs transplant therapy in rat uterus injury

**Figure 3:**
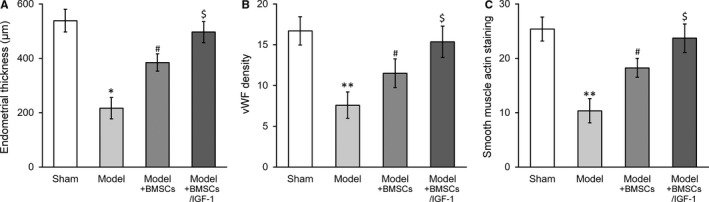
IGF‐1 stable expression further enhances BMSCs transplant therapy in treating injured rat uterus. Rats were divided into sham, model, model receiving control BMSC transplant (Model + BMSCs) and model receiving IGF‐1‐transduced BMSC transplant (Model + BMSCs/IGF‐1), with 8 rats per group.Twenty‐eight days after the treatment, (A) thickness in the regenerative uterine horns, (B) neovascularization measured as vWF staining density in the newly regenerated uterine endometrial and (C) smooth muscle regeneration measured as smooth muscle actin staining, respectively, were assayed in the experimental animals. Values are mean ± SD (n = 8 each group). **P < .01, *P < .05, vs sham. ^#^ *P* < .05 vs both sham and model. ^$^ *P* < .05 vs both model and model+BMSCs. IGF‐1, Insulin‐like growth factor‐1; BMSC, Bone marrow‐derived mesenchymal stem cells

Next, we sought to investigate the potential therapeutic effect of IGF‐1‐overexpressed BMSC transplantation in rat uterus injury model. The model was successfully established following the previously described method. The regeneration of injured uterus was histologically examined, and the thickness was measured at 28 days after surgery. As shown in Figures 3A and S2A, the endometrial thickness in the model group was dramatically lower than the sham control rats, which indicated the well‐recapitulated pathologic processing of endometrium impairment in our system. Transplantation with BMSCs by injection into the rat uterus significantly increased the endometrial thickness, which further consolidated the efficacy of stem cell therapy for this complication. Notably, the IGF‐1 overexpressing BMSCs cells displayed the remarkable improvement in this setting while in comparison with regular BMSCs transplantation and almost led to complete restoration of the endometrial thickness.

In addition, we evaluated the neovascularization and smooth muscle regeneration during recovery phase via immunostaining with specific markers as well. The angiogenesis indicated by vWF labelling was significantly induced post‐BMSCs transplantation, which was further enhanced by overexpression of IGF‐1 (Figures 3B and S2B). Similarly, the smooth muscle regeneration, which was greatly impaired in model group, was greatly improved by IGF‐1 in BMSCs transplantation (Figures 3C and S2C). Our in vivo data supported the beneficial effect of IGF‐1 in promotion recovery of injured uterus.

### 3.4 Inhibition of NF‐κB p50 subunit abolishes IGF‐1‐induced expression and secretion of IL‐10 in stable IGF‐1‐expressing BMSCs

**Figure 4:**
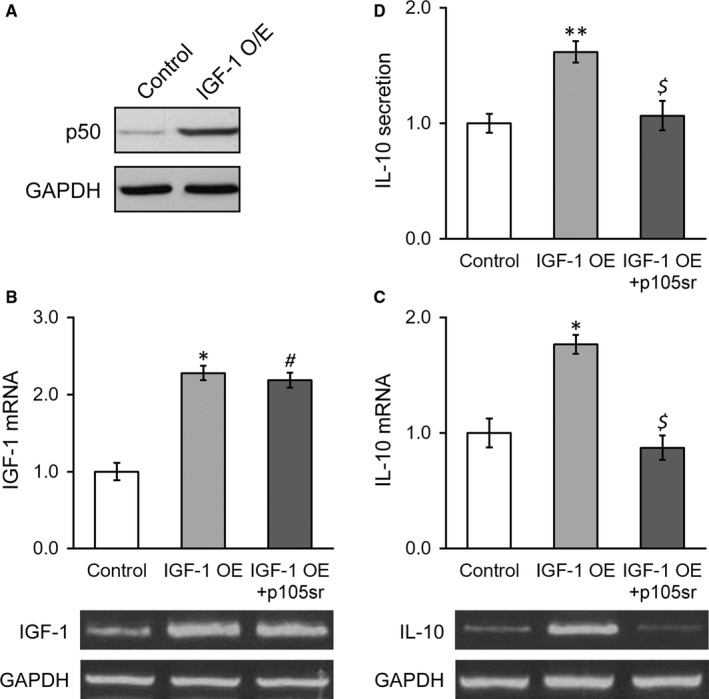
Inhibition of NF‐κB p50 subunit abolishes IGF‐1‐induced expression and secretion of IL‐10 in stable IGF‐1‐expressing BMSCs. (A) p50 protein level was examined in control BMSCs and IGF‐1‐transduced BMSCs. B and C, mRNA levels of IGF‐1 (B) and IL‐10 (C) were examined in control BMSCs, IGF‐1‐transduced BMSCs and IGF‐1‐transduced BMSCs treated with p105sr, with representative gel images of PCR products shown below the bar graph. (D) IL‐10 levels in media culturing control BMSCs, IGF‐1‐transduced BMSCs and IGF‐1‐transduced BMSCs treated with p105sr. Values are mean ± SD. **P < .01, *P < .05, vs control. ^#^ *P* < .05 vs control and not significant vs IGF‐1 OE. $ not significant vs control and *P* < .05 vs IGF‐1 OE. IGF‐1, Insulin‐like growth factor‐1; BMSC, Bone marrow‐derived mesenchymal stem cells

Our previous results suggested that overexpression of IGF‐1 in BMSCs improved its therapeutic effect in rat uterus injury model.^16^ Next, we attempted to elucidate the detailed molecular events underlying this process. It is reported that NF‐κB signalling might involve in IL‐10 regulation, which prompted us to examine the change of p50 subunit and potential contribution to IL‐10 modulation in response to IGF‐1 overexpression. As shown in Figure 4A, the protein level of p50 was significantly induced in IGF‐1 overexpressed BMSCs cells in comparison with control, which suggested the activation of NF‐κB pathway and the potential involvement in IL‐10 regulation. To address this issue, here we employed p50‐specific inhibitor, p105sr, to treat IGF‐1‐overexpressed BMSCs cells. Results from the RT‐PCR experiments demonstrated that p105sr treatment imposed no influence on IGF‐1 expression, which indicated that p50 functioned downstream IGF‐1 along this signalling transduction pathway (Figure 4B). However, both expression at transcriptional level and secretion of IL‐10 were markedly compromised by p50sr treatment (Figure 4C,D), which highlighted the predominant role of NF‐κB signalling in IL‐10 regulation by IGF‐1.

### 3.5 Knockdown of NF‐κB p50 subunit abolishes IGF‐1‐induced expression and secretion of IL‐10 in stable IGF‐1‐expressing BMSC

**Figure 5:**
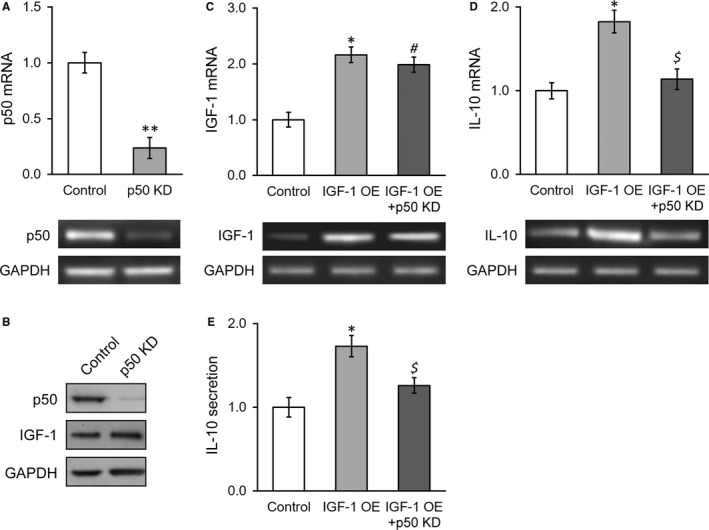
Knockdown of NF‐κB p50 subunit abolishes IGF‐1‐induced expression and secretion of IL‐10 in stable IGF‐1‐expressing BMSCs. (A) p50 mRNA levels were examined in control BMSCs and p50 knockdown BMSCs, with representative gel images of PCR products shown below the bar graph. (B) Protein levels of p50 and IGF‐1 were examined in control BMSCs and p50 knockdown BMSCs. C and D, mRNA levels of IGF‐1 (C) and IL‐10 (D) were examined in control BMSCs, IGF‐1‐transduced BMSCs and IGF‐1‐transduced BMSCs with p50 knockdown, with representative gel images of PCR products shown below the bar graph. (E) IL‐10 levels in media culturing control BMSCs, IGF‐1‐transduced BMSCs and IGF‐1‐transduced BMSCs with p50 knockdown. Values are mean ± SD. **P < .01, *P < .05, vs control. ^#^ *P* < .05 vs control and not significant vs IGF‐1 OE. ^$^Not significant vs control and *P* < .05 vs IGF‐1 OE. IGF‐1, Insulin‐like growth factor‐1; BMSC, Bone marrow‐derived mesenchymal stem cells

We further consolidated our previous observation, and NF‐κB p50 subunit was specifically knocked down by shRNA. The knockdown efficiency was first validated by RT‐PCR as shown in Figure 5A and immunoblotting in Figure 5B. The inhibition of p50 did not affect IGF‐1 at both transcript and protein levels, which was consistent with the results from p50sr‐treated BMSCs (Figure 5B,C). In contrast, shRNA‐mediated knockdown of p50 almost entirely abolished IGF‐1‐induced expression and secretion of IL‐10 in BMSCs cells (Figure 5D,E), which unambiguously demonstrated that p50 functioned downstream IGF‐1 in up‐regulation IL‐10.

### 3.6 NF‐κB p50 subunit induces IL‐10 expression by directly binding to its promoter region

**Figure 6:**
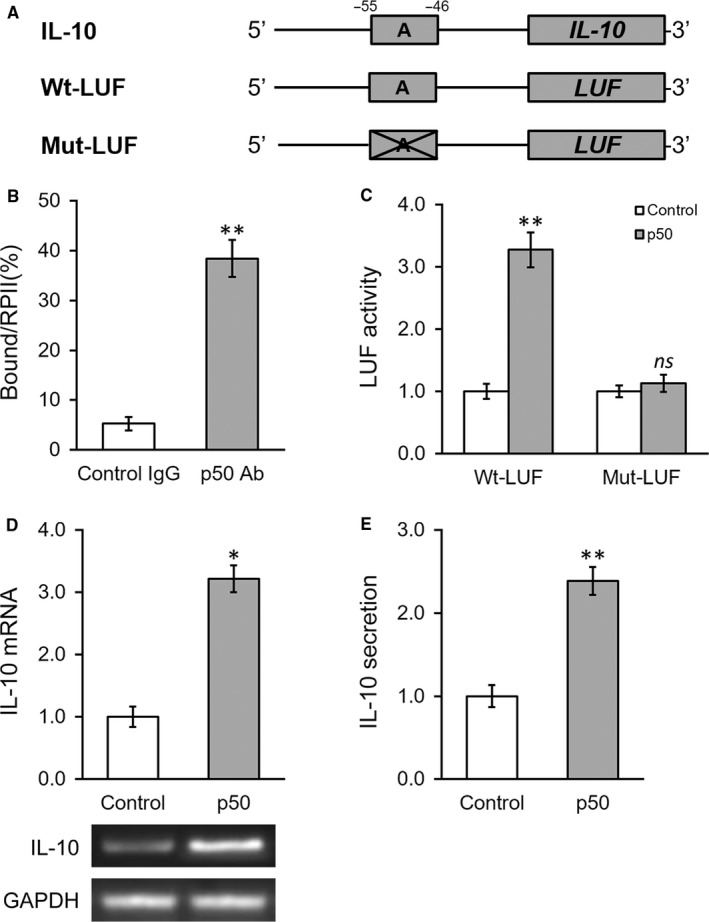
NF‐κB p50 subunit induces IL‐10 expression by directly binding to its promoter region. A, The putative p50 binding site was identified in the promoter region of IL‐10. Wild‐type (Wt‐LUF) and mutated (Mut‐LUF) sequences from IL‐10 promoter were cloned, respectively, to the 5′‐prime of luciferase reporter gene (LUF). B, Binding of p50 to the putative binding site in IL‐10 promoter was analysed by ChIP assay using control IgG and p50 antibody (Ab), respectively. C, Luciferase activities of Wt‐LUF or Mut‐LUF constructs described in (A) were measured in BMSCs transfected with control vector or p50, respectively. D and E, Levels of IL‐10 mRNA with representative gel images of PCR products shown below the bar graph (D), and in media (E) were examined in BMSCs transfected with control vector or p50, respectively. Values are mean ± SD. **P < .01, *P < .05, vs respective control

Our previous results showed the critical role of p50 in induction of IL‐10 expression and secretion by IGF‐1, which the underlying molecular mechanism was still to be defined. The close inspection of IL‐10 promoter sequence has identified a proximal putative binding site of p50 as illustrated in Figure 6A. The direct binding of p50 to the predicted sequence was experimentally confirmed by ChIP assay, which showed around sevenfold enrichment of p50‐responsive element sequence in p50‐immunoprecipitate in comparison with IgG control (Figure 6B). Furthermore, we constructed either wild‐type or mutated IL‐10 promoter‐driven luciferase reporter plasmids to intuitively investigate the regulatory effect of p50 on IL‐10 (illustrated in Figure 6A). As shown in Figure 6C, the ectopic expression of p50 significantly stimulated the luciferase activity in comparison with control, while the mutations introduced in p50‐responsive elements readily abrogated this inducing effect. Likewise, the forced expression of p50 in BMSCs cells induced significant up‐regulation of endogenous IL‐10 expression and secretion (Figure 6D,E). Our data clearly demonstrated that p50 activated transcription of IL‐10 in BMSCs in response to IGF‐1 overexpression via direct association with its promoter and consequently promoted the anti‐inflammatory cytokine secretion and functioning.

### 3.7 Enhancing effect of IGF‐1 stable expression on BMSCs transplant therapy in treating injured rat uterus requires p50

**Figure 7:**
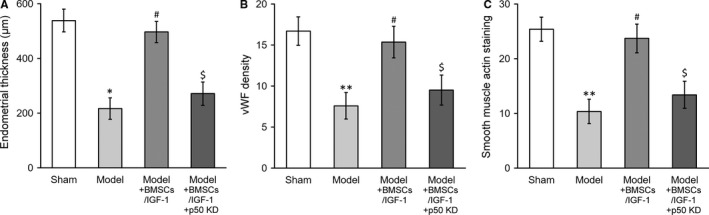
Enhancing effect of IGF‐1 stable expression on BMSCs transplant therapy in treating injured rat uterus requires p50. Rats were divided into sham, model, model receiving IGF‐1‐transduced BMSC transplant (Model+BMSCs/IGF‐1) and model receiving IGF‐1‐transduced and p50 knockdown BMSC transplant (Model+BMSCs/IGF‐1+p50 KD), with 8 rats per group. Twenty‐eight days after the treatment, (A) thickness in the regenerative uterine horns, (B) neovascularization measured as vWF staining density in the newly regenerated uterine endometrial and (C) smooth muscle regeneration measured as smooth muscle actin staining, respectively, were assayed in the experimental animals. Values are mean ± SD (n = 8 each group). **P < .01, *P < .05, vs sham. ^#^ *P* < .05 vs model. ^$^ *P* < .05 vs both sham and Model+BMSCs/IGF‐1. IGF‐1, Insulin‐like growth factor‐1; BMSC, Bone marrow‐derived mesenchymal stem cells

Our previous data highlighted the indispensable role of p50 in mediating IGF‐1‐induced up‐regulation and secretion of IL‐10. Both inhibition and knockdown of p50 remarkably abolished IGF‐1‐elicited expression and secretion of IL‐10 in vitro. Next, we sought to validate this in vivo to exclude the potential artefacts associated with cell culture. To this purpose, here we established p50 stable knockdown cell line in IGF‐1‐overexpressed BMSCs. In sharp contrast to its parental cell line, p50 deficiency almost completely compromised the IGF‐1‐imposed stimulatory effect with respect to endometrial thickness (Figures 7A and S3A). Likewise, the angiogenesis induced by transplantation of IGF‐1‐overexpressed BMSCs was markedly suppressed by p50 silencing (Figures 7B and S3B). The smooth muscle regeneration indicated by α‐SMA immunostaining was reduced in p50‐deficient group in comparison with scramble control as well (Figures 7C and S3C). Our data consolidated that p50 was indispensable for IGF‐1‐conferred therapeutic effect of BMSCs transplantation.

## 4 DISCUSSION

The severe endometrial damage frequently associates with amenorrhoea and infertility, which imposes huge burden on women both physically and psychologically.^19^ With the limitation of available clinical options for this complication, the exploitation focus has been increasingly concentrated on BMSCs‐based therapy. To circumvent the inherent barrier regarding the rarity in bone marrow and compromised differentiative potential, here we introduced IGF‐1 into BMSCs, which significantly improved BMSCs transplantation therapy in rat uterus injury model. The previous study suggested that IGF‐1 could suppress the inflammatory cytokines release and inhibit the excessive inflammation and fibrous scar formation in endometrial injury.^12^ In agreement with this observation, here we further demonstrated that IGF‐1 overexpression significantly stimulated the production and secretion of IL‐10 in BMSCs cells, which was synergistically enhanced by controlled administration with oestrogen nanoparticles. Our results defined a mode of action that gene‐engineered BMSCs held great promise for therapeutic purpose against uterus injury. However, the molecular mechanism underlying IGF‐1‐stimulated expression and secretion of IL‐10 was still elusive in this setting.

To gain better insight into the beneficial effect of IGF‐1 overexpression in BMSCs transplantation, here we attempted to understand the regulatory model of IGF‐1 on transcription of IL‐10. Aided by available bioinformatics online tools and close inspection of the IL‐10 proximal promoter sequence, we have identified the putative p50 recognition site and binding to this motif was further experimentally validated through ChIP assay. As a transcriptional factor per se, the association of p50 with its *cis‐*element immediately driven downstream gene transcription as exemplified by artificial luciferase and endogenous IL‐10. Consistently, Warzecha et al demonstrated that IGF‐1 stimulated production of IL‐10 and inhibited development of caerulein‐induced pancreatitis.^20^ Lu et al showed that addition of recombinant IGF‐1 stimulated IL‐10 production by activated normal T cells, which was impaired by subsequent transfection with miR‐223 mimic.^21^ Strle et al reported that prototypical anti‐inflammatory cytokine IL‐10 prevented loss of IGF‐1‐induced myogenin protein expression caused by IL‐1beta.^22^ Kooijman et al suggested that IGF‐1 might exert inhibitory actions on inflammatory and Th1‐mediated cellular immune responses through stimulation of IL‐10 production in T cells.^23^ Along with our results, all these above‐mentioned observations implicated that IGF‐1 might increase IL‐10 expression and secretion in a wide range of physiological and pathological conditions.

Our data highlighted the essential and predominant role of NF‐κB signalling, especially the p50 subunit, to function downstream IGF‐1 and activate IL‐10 transcription. Cumulative evidence consolidated our observations that NF‐κB regulated IL‐10 expression in variety of cell types. For instance, Wei et al showed dual‐parallel inhibition of IL‐10 and TGF‐β1 controlled LPS‐induced inflammatory response via NF‐κB signalling in monocytes/macrophages.^24^ Leghmari et al demonstrated that HIV‐1 Tat protein induced IL‐10 production in monocytes by classical and alternative NF‐κB pathways.^25^ Liu et al indicated that LPS‐induced transcriptional activation of IL‐10 was mediated by MAPK‐ and NF‐κB‐induced CCAAT/enhancer‐binding protein delta in mouse macrophages.^26^ Dibra et al demonstrated that the cell‐to‐cell co‐ordination between activated T cells and CpG‐stimulated macrophages synergistically induced elevated levels of IL‐10 via NF‐κB, STAT3 and CD40/CD154.^27^ Furthermore, our data particularly emphasized the predominant role of p50 subunit in activation of IL‐10, which was in agreement with previous study presented by Cao et al suggesting that p50 homodimers specifically bound to ‐55/‐46 locus in the promoter and induced IL‐10 expression.^28^ Conversely, Tomczak et al showed that the ability of IL‐10 to control LPS‐induced expression of IL‐12 was significantly compromised in p50/p105 deficient macrophages.^29^ These data suggested that in response to overexpression of IGF‐1 in the inflammatory milieu, NF‐κB pathway was subsequently activated and p50 transcriptional factor translocated into nucleus, which specifically bound to IL‐10 promoter region and activated transcription. The stimulated production and secretion of anti‐inflammatory cytokine IL‐10 consequently ameliorated inflammatory reactions.

Noteworthily, how the NF‐κB pathway was activated by IGF‐1 in BMSCs was not addressed in detail in our current study. It is well‐acknowledged that insulin/IGF‐1 receptor signalling activated the PI‐3K/AKT kinase cascade which in turn influence several elementary downstream pathways, especially AKT could stimulate the NF‐κB signalling via the activation of IKK complex. For example, Iwasaki et al suggested that regular insulin treatment enhanced the MAPK‐dependent phosphorylation of NF‐κB components and IKK‐dependent nuclear translocation of NF‐κB complexes, which convergently contributed to the NF‐κB pathway activation.^30^ Mitsiades et al demonstrated activation of NF‐κB and up‐regulation of intracellular anti‐apoptotic proteins via the IGF‐1/Akt signalling in human multiple myeloma cells.^31^ Whereas in endothelial cells, Che et al indicated that IGF‐1 enhanced inflammatory responses via negative regulation of Gab1 and MEKK3 and hence positive activation of c‐Jun and NF‐κB.^32^ Therefore, the molecular signalling pathway downstream IGF‐1 to activate NF‐κB is still to be defined in our gene‐engineered BMSCs cells.

In summary, here we successfully established endometrial damage rat model to precisely recapitulate the pathological process and spontaneous recovery phase. Our results unambiguously demonstrated that gene‐engineered IGF‐1‐BMSCs significantly improved transplantation therapy in comparison with mock treatment. Our strategy provided a way to optimize the clinical effect of BMSCs transplantation for the future exploitations. Mechanistically, overexpressed IGF‐1 activated NF‐κB pathway, which in turn induced IL‐10 expression via direct binding of p50 to its promoter. The elevated production and secretion of anti‐inflammatory cytokine IL‐10 eventually contributed to inflammation elimination and endometrial regeneration. Further investigations with respect to clinical application are warranted.

## CONFLICT OF INTEREST

There is no conflict of interest that could be perceived as prejudicing the impartiality of the research reported.
