# Supplementary material for: Transplant of insulin‐like growth factor‐1 expressing bone marrow stem cells improves functional regeneration of injured rat uterus by NF‐κB pathway
Source: J Cell Mol Med. 2018 Mar 7;22(5):2815–25. doi: 10.1111/jcmm.13574 (PMC5908117; doi:10.1111/jcmm.13574)
Supplement: Supplementary file 1 [file JCMM-22-2815-s001.docx]

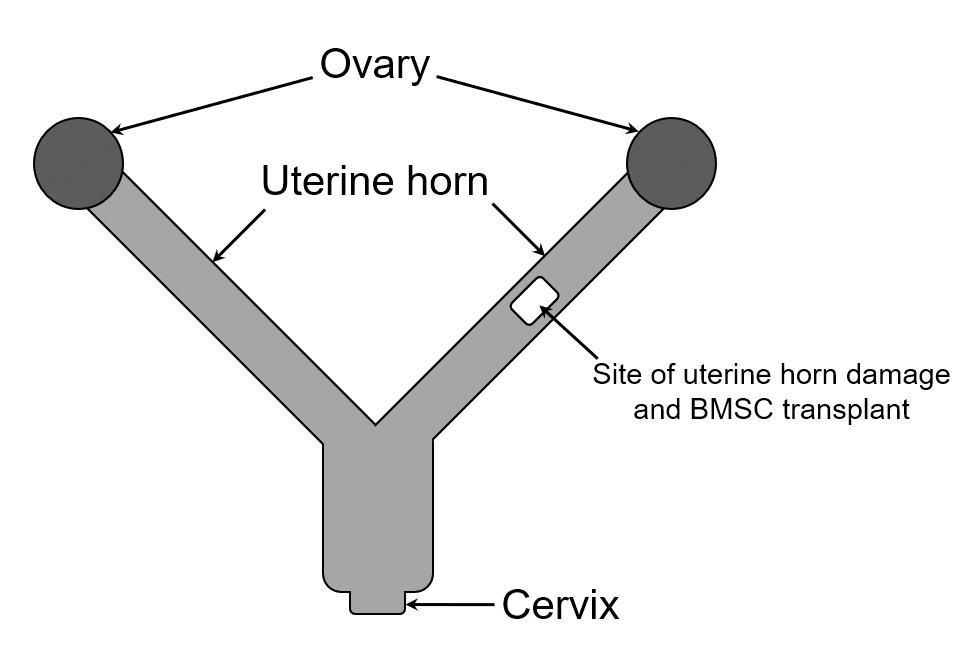


**Figure S1. Illustration of BMSC transplant into the rat uterine horn damage model.**


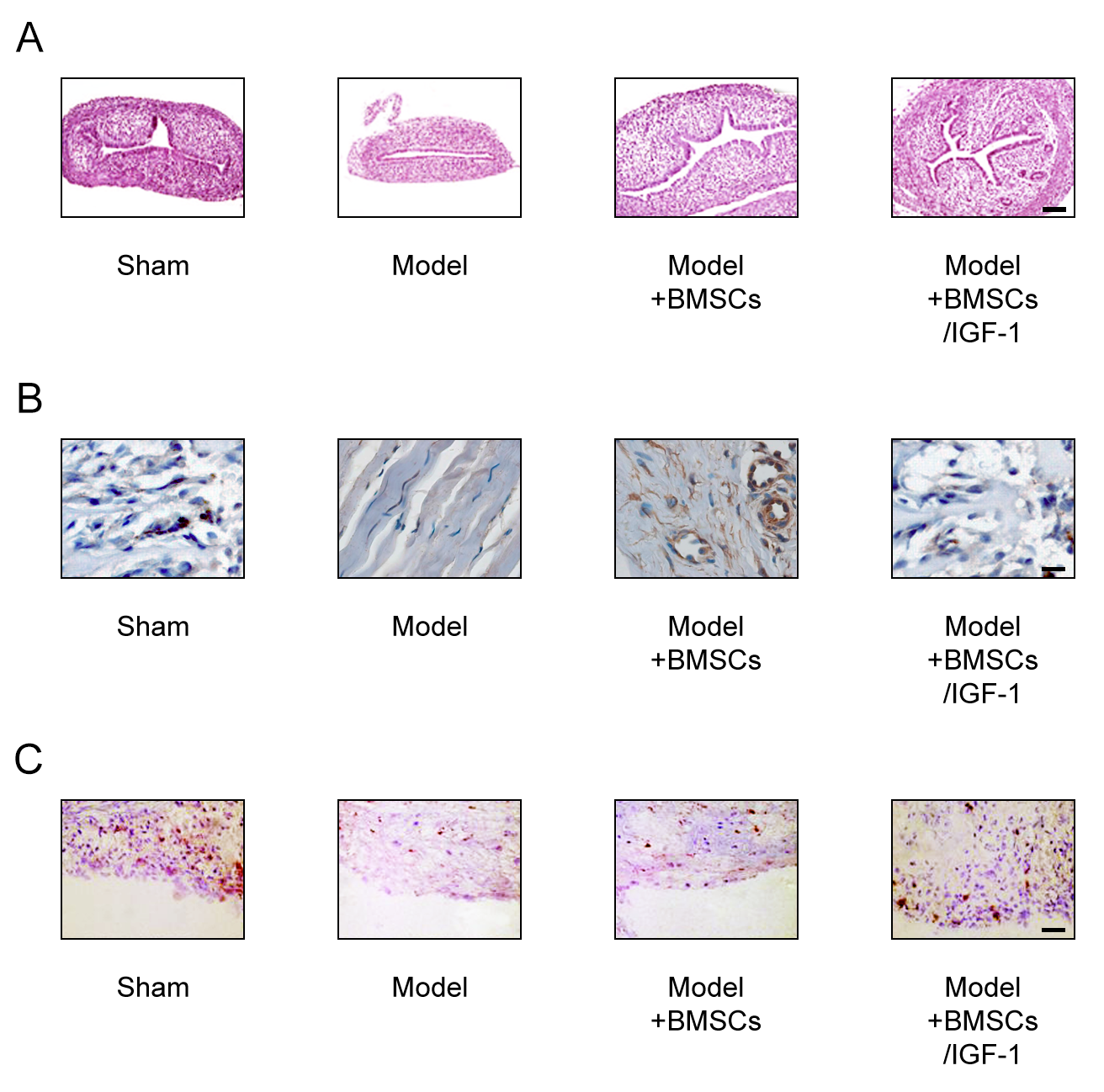


**Figure S2. IGF-1 stable expression further enhances BMSCs transplant therapy in treating injured rat uterus.**

Rats were divided into sham, model, model receiving control BMSC transplant (Model+BMSCs) and model receiving IGF-1 transduced BMSC transplant (Model+BMSCs/IGF-1), with 8 rats per group. 28 days after the treatment, representative histological images of (A) thickness in the regenerative uterine horns (scale bar 200 μm), (B) neovascularization measured as vWF staining density in the newly regenerated uterine endometrial (scale bar 20 μm) and (C) smooth muscle regeneration measured as smooth muscle actin staining (scale bar 50 μm), respectively, were shown.


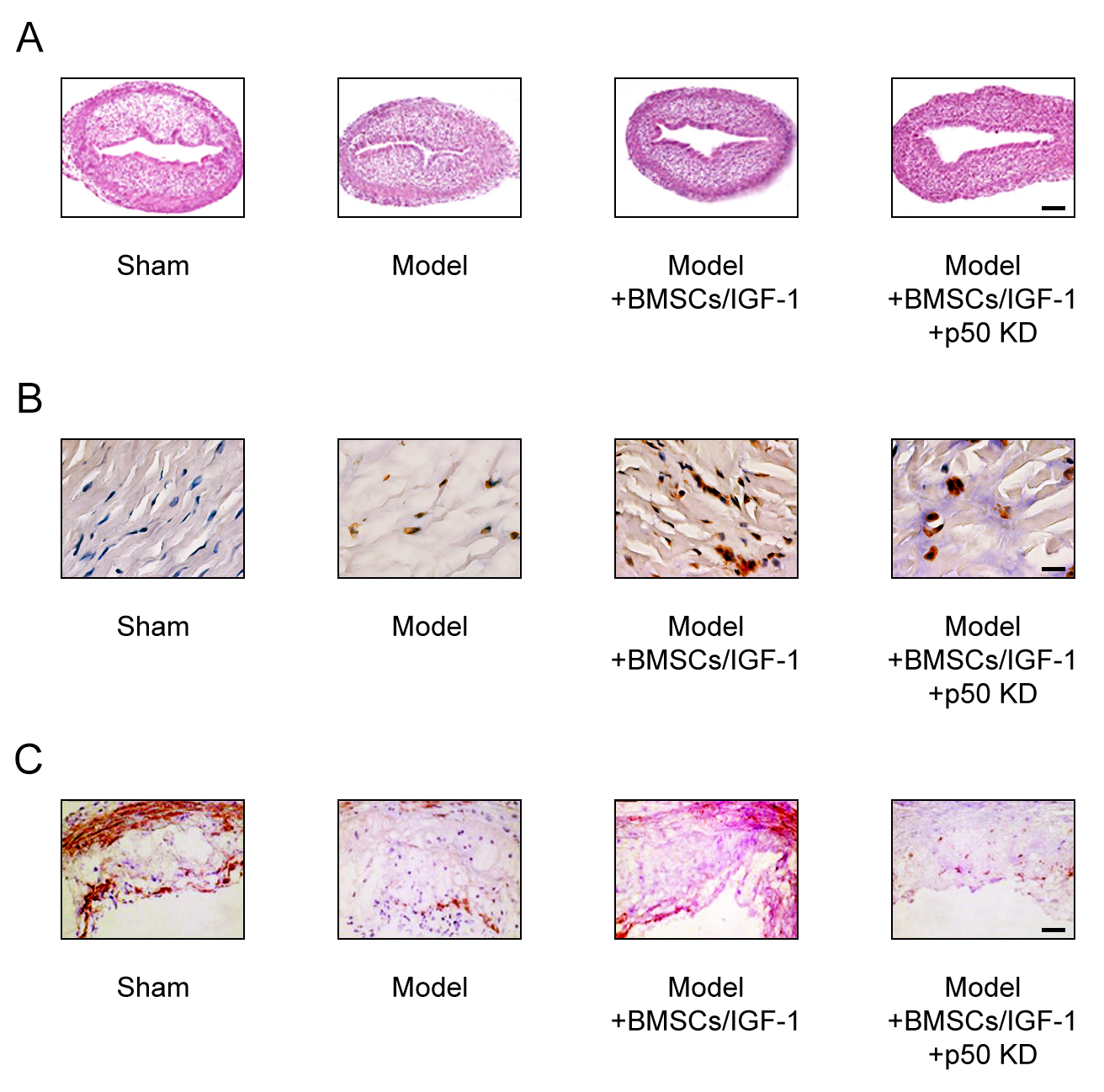


**Figure S3. Enhancing effect of IGF-1 stable expression on BMSCs transplant therapy in treating injured rat uterus requires p50.**

Rats were divided into sham, model, model receiving IGF-1 transduced BMSC transplant (Model+BMSCs/IGF-1) and model receiving IGF-1 transduced and p50 knockdown BMSC transplant (Model+BMSCs/IGF-1+p50 KD), with 8 rats per group. 28 days after the treatment, representative histological images of (A) thickness in the regenerative uterine horns (scale bar 200 μm), (B) neovascularization measured as vWF staining density in the newly regenerated uterine endometrial (scale bar 20 μm) and (C) smooth muscle regeneration measured as smooth muscle actin staining (scale bar 50 μm), respectively, were shown.
